# Extensive daily movement rates measured in territorial arctic foxes

**DOI:** 10.1002/ece3.7165

**Published:** 2021-01-26

**Authors:** Marie‐Pier Poulin, Jeanne Clermont, Dominique Berteaux

**Affiliations:** ^1^ Canada Research Chair on Northern Biodiversity and Center for Northern Studies Université du Québec à Rimouski Rimouski QC Canada

**Keywords:** fix interval, GPS satellite tracking, home range, movement rate, traveled distance, *Vulpes lagopus*

## Abstract

An animal's movement rate is a central metric of movement ecology as it correlates with its energy acquisition and expenditure. Obtaining accurate estimates of movement rate is challenging, especially in small highly mobile species where GPS battery size limits fix frequency, and geolocation technology limits positions’ precision. In this study, we used high GPS fix frequencies to evaluate movement rates in eight territorial arctic foxes on Bylot Island (Nunavut, Canada) in July–August 2018. We also assessed the effects of fix interval and location error on estimated movement rates. We obtained 96 fox‐days of data with a fix interval of 4 min and 12 fox‐days with an interval of 30 s. We subsampled the latter dataset to simulate six longer fix intervals ranging from 1 to 60 min and estimated daily distances traveled by adding linear distances between successive locations. When estimated with a fix interval of 4 min, daily distances traveled by arctic foxes averaged 51.9 ± 11.7 km and reached 76.5 km. GPS location error averaged 11 m. Daily distances estimated at fix intervals longer than 4 min were greatly underestimated as fix intervals increased, because of linear estimation of tortuous movements. Conversely, daily distances estimated at fix intervals as small as 30 s were likely overestimated due to location error. To our knowledge, no other territorial terrestrial carnivore was shown to routinely travel daily distances as large as those observed here for arctic foxes. Our results generate new hypotheses and research directions regarding the foraging ecology of highly mobile predators. Furthermore, our empirical assessment of the effects of fix interval and location error on estimated movement rates can guide the design and interpretation of future studies on the movement ecology of small opportunistic foragers.

## Graphical abstract

In this study, we used high GPS fix frequencies to evaluate movement rates in eight territorial arctic foxes on Bylot Island (Nunavut, Canada) in July–August 2018. We demonstrated that arctic foxes are highly mobile predators that travel extensive distances daily within their summer territories. Our study also stresses the need to use an optimal sampling frequency when measuring animal movement, since fixes obtained at long intervals greatly underestimate movement rates of highly mobile animals.

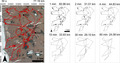

## 1 INTRODUCTION

Movements of organisms correlate with many biological processes at the individual and population levels, such as energy expenditure, habitat selection, and population dynamics (Brown & Orians, 1970; Morales et al., 2010). Studying the characteristics of movement can thus answer important ecological questions such as why, how, when, and where mobile organisms change location. Understanding movements is also key to unravel community and ecosystem processes such as predator–prey interactions (Kays et al., 2015; Nathan, 2008). For example, predator space use influences prey behavior through nonconsumptive effects (Fortin et al., 2005; Laundré, 2010), and antipredator responses can have far‐reaching ecological consequences (Boonstra et al., 1998).

The activity and movement characteristics of predators are used to classify their foraging tactics along a continuum ranging from active predators to sit‐and‐wait predators (Avgar et al., 2008; Huey & Pianka, 1981; Scharf et al., 2006). Active predators are highly mobile and usually have a higher encounter rate with their prey than sit‐and‐wait predators, who ambush them (Avgar et al., 2008; Scharf et al., 2006). Distance traveled per unit of time (or movement rate) is a strong predictor of predator searching efficiency (Holling, 1959; Merrill et al., 2010). For example, daily distances traveled by gray seals (*Halichoerus grypus*) predict their feeding frequency at different temporal scales (Austin et al., 2006). As a result, distances traveled by predators may influence prey fitness. For instance, black‐capped vireo (*Vireo atricapilla*) nest survival is negatively correlated with Texas ratsnake (*Elaphe obsoleta*) daily distance traveled (Sperry et al., 2008).

Modern tracking technologies, such as the Global Positioning System (GPS), now allow measurement of animal movement at high resolution (Hofman et al., 2019; Kays et al., 2015; Wilmers et al., 2015). In addition, hardware miniaturization opens many new research opportunities. Longer battery life and solar recharging capabilities also allow higher fix frequencies (Hofman et al., 2019), thus decreasing biases in estimation of distances traveled by animals (Joly, 2005; Rowcliffe et al., 2012; Mills et al., 2006; Noonan et al., 2019; Pépin et al., 2004).

Underestimation of distances traveled by animals is a recurrent problem in tracking studies, as estimates are made by adding linear distances between locations, even though most movements are tortuous (Rowcliffe et al., 2012; Noonan et al., 2019). Yet, this underestimation is rarely quantified (Rowcliffe et al., 2012). Of the few studies addressing this problem, Rowcliffe et al. (2012) found a strong relationship between tortuosity, sample frequency, and distance traveled for 10 terrestrial mammal species in Panama. Pépin et al. (2004) found that the number of attempted fixes per day predicted estimated distances traveled by red deer (*Cervus elaphus*). Sampling frequency also affected estimates of territory size and distances moved by the eastern timber wolf (*Canis lycaon*), with the latter decreasing exponentially with the reduction of sampling frequency (Mills et al., 2006). Not using an optimal sampling frequency when measuring animal movement can reduce the robustness of hypothesis testing, either because too low sampling frequencies underestimate movement rates, or too high sampling frequencies reduce the sampling period (if battery depletion shortens the study) or the sample size of tracked animals (if battery depletion forces the sequential use of multiple sensors on the same individuals rather than their simultaneous use on different individuals). The fact that estimates of distances traveled by animals are affected by sampling frequency, movement tortuosity, and location error (Jerde & Visscher, 2005; Swain et al., 2008) thus requires detailed investigations. As an example, Noonan et al. (2019) recently outlined a continuous time distance estimation method to provide scale‐insensitive estimates of tracked animals, and this method offered dramatic improvements over typical straight line displacement summation methods when estimating distance traveled by a reptile and a mammal tracked at 60‐min and 15‐min intervals, respectively.

Interestingly, the greater capacity of batteries and their solar recharging capabilities, which allow higher fix frequencies, could also lead to overestimation of distances traveled by animals. Indeed, if fixes are so frequent that movements of animals between fixes are small compared to location error, then estimated traveled distances will reflect location error more than true movement (Jerde & Visscher, 2005). Assessing this potential problem requires knowledge of GPS location error, of the relation between GPS location error and fix frequency, and of movement rates of the studied animals.

**FIGURE 1:**
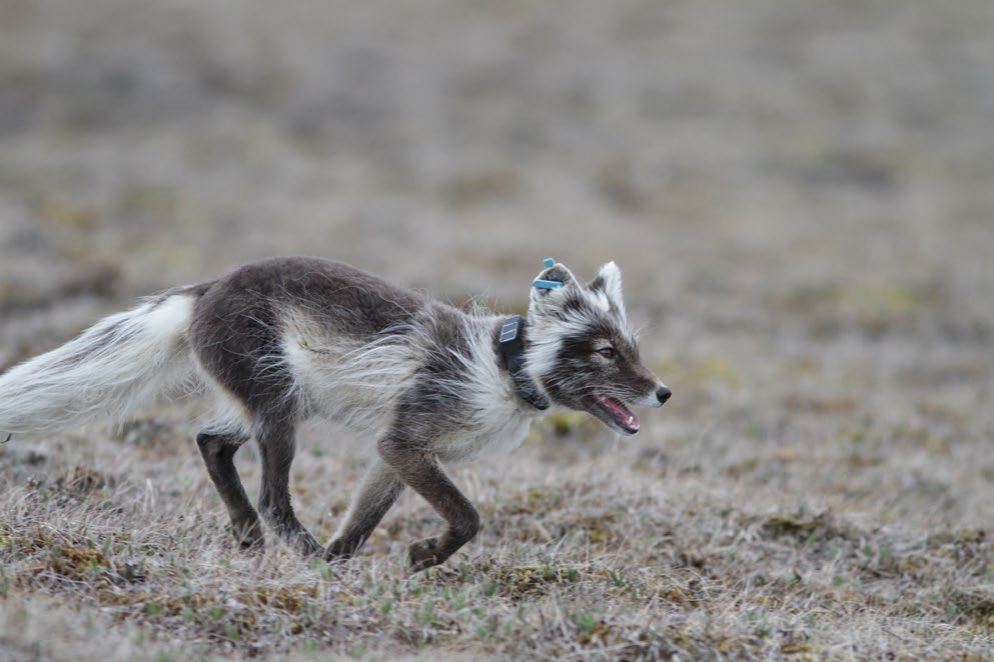
Arctic fox in summer pelage wearing ear tags and a GPS collar equipped with a solar panel and a rechargeable battery (7 July 2018, Bylot Island, Nunavut, Canada). Photo credit: Dominique Berteaux

The arctic fox (*Vulpes lagopus*; Figure 1) is a territorial circumpolar opportunistic predator (Eberhardt et al., 1982; Eide et al., 2005) with top‐down effects on the tundra ecosystem (Legagneux et al., 2012). Extensive movements of 90 km/day (Tarroux et al., 2010), 112 km/day (Lehner, 2012), and 150 km/day (Fuglei & Tarroux, 2019) have been estimated with the Argos system for dispersing individuals. While this demonstrates the physical ability of this small terrestrial carnivore for long‐distance traveling on snow and ice during dispersal events, fine‐scale movements inside the summer territory are largely unknown.

Our primary objective was to estimate the intensity of arctic fox movements within their summer territories, using a fix frequency allowing virtually unbiased estimates of distances traveled by individuals. We predicted that daily distances traveled within territories in summer by arctic foxes would be larger than those published to date in most other species, given (a) the demonstrated high motion capacity of arctic foxes, (b) the benefits that mobile predators incur when they increase prey encounter rate, and (c) the underestimation of daily traveled distances obtained to date in most telemetry studies. A secondary (methodological) objective was to assess empirically the effects of fix interval and location error on daily distance traveled by individuals. Based on the literature (Jerde & Visscher, 2005; Joly, 2005; Rowcliffe et al., 2012; Mills et al., 2006; Noonan et al., 2019; Pépin et al., 2004; Swain et al., 2008), we predicted that estimated distances traveled daily should be logarithmic and exponential functions of fix frequency and location error, respectively.

## 2 METHODS

### 2.1 Study area

We worked in the southern plain of Bylot Island (73°N, 80°W), which is part of Sirmilik National Park of Canada, Nunavut. The arctic fox is the main terrestrial predator of the island (Gauthier et al., 2011). The brown (*Lemmus trimucronatus*) and the collared lemming (*Discrotonyx groenlandicus*) are the primary prey of arctic foxes (Gauthier et al., 2011). Brown lemmings, the most abundant species, show large amplitude cycles with a periodicity of 3–4 years (Gruyer et al., 2008) and predation by arctic foxes and birds limits lemming population growth during summer (Fauteux et al., 2016). The southern plain of the island also encompasses a large but spatially restricted greater snow goose (*Chen caerulescens atlantica*) breeding colony (Bêty et al., 2001). Other migratory birds nest at lower densities in the area during summer (Lepage et al., 1998). Arctic foxes prey on migratory birds, primarily goose eggs and chicks, especially when rodent populations are low (Bêty et al., 2001; McKinnon et al., 2013). Lemming density was low in summer 2018 (0.01 lemming/ha; D. Fauteux, pers. comm.). The bird nesting period (early June to mid‐July) coincides with an intensive period of arctic fox parental care, when cubs need to be provided with both milk and solid food at their den.

### 2.2 Capture and satellite tracking

From 3 June to 2 July 2018, we captured four adult males (M1 to M4) and four adult females (F1 to F4), using Tomahawk cage traps #205 (Tomahawk Live Trap Company) or Softcatch #1 padded leghold traps (Oneida Victor Inc. Ltd.) in the goose colony. Capture techniques and immobilization procedures were approved by the UQAR Animal Care Committee (CPA‐64‐16‐169 R2), and field research was approved by the Joint Park Management Committee of Sirmilik National Park of Canada (SIR‐2018‐8021). Table 1 provides details about the life history of captured individuals. Each fox was fitted with a GPS collar (95 g, 2.6%–3.3% of body mass; Radio Tag‐14, Milsar Technologies S.R.L.; Figure 1). Our GPS collars were equipped with a rechargeable battery, a solar panel, and UHF transmission allowing remote data download and reprogramming. According to manufacturer specifications, location error of GPS units is independent of fix interval at fix intervals > 7 s, when tracking of satellite signals is intermittent, but decreases at shorter intervals, because units then keep track of visible satellite signals continuously (P. Otulak, Milsar Technologies S.R.L., pers. comm.). From 5 to 16 July (12 days), we set GPS fix interval of all collars to 4 min and obtained 96 fox‐days of data. During this period, fox cubs require high parental investment while geese transition from their incubation to brooding stages. From 29 July to 2 August (5 days), we set fix interval of 3 collars (M1, F1, F2) to 30 s and obtained 12 fox‐days of data (3 fox‐days were discarded because > 911 (31.63%) of 2,880 potential fixes were unsuccessful, likely because foxes spent time in their den). During this period, fox cubs gradually become independent from their parents while goose chicks are about halfway between birth and fledging.

**TABLE 1:** Individual characteristics of eight arctic foxes GPS‐tracked in 2018 on Bylot Island (Nunavut, Canada)

Fox ID	Pair	Sex	Capture date	Mass (kg)	Age class	Status	Color code	Unique ID
F1		F	14 Jun 2018	3.20	Prime‐age adult	R	JVOJ	717
F2	A	F	02 Jul 2018	3.25	Prime‐age adult	NR	BVOB	746
F3	B	F	05 Jun 2018	2.85	Prime‐age adult	R	OJOO	722
F4	C	F	22 Jun 2018	2.88	Old	R	JMVJ	376
M1		M	08 Jun 2018	3.05	Prime‐age adult	NR	ORRR	743
M2	C	M	03 Jun 2018	3.45	Prime‐age adult	R	OBBB	718
M3	B	M	05 Jun 2018	3.65	Prime‐age adult	R	RVJO	737
M4	A	M	02 Jul 2018	3.34	Prime‐age adult	NR	JBOR	747

Note

All foxes captured in the study area received a color code for field identification and a unique identifier for data management, while those used in this study were also given a short identifier (Fox ID) to ease references in the text. Foxes belonging to the same pair shared the same territory. Capture date indicates when each fox was captured, weighed and fitted with a GPS collar. Age class (Prime‐age adult = 2–4 years old, Old = ≥5 years old) was determined from tooth wear (Chevallier et al., 2017). Reproductive status (R = Reproductive, NR = Nonreproductive) indicates whether some cubs were observed (automated cameras, see Cameron et al., 2011) at the individual's den in 2018.

### 2.3 Data processing and analyses

We removed from analyses all fixes obtained < 48 hr after capture and handling. We assessed precision of fixes using Horizontal Dilution of Precision (HDOP) (D'Eon & Delparte, 2005; Lewis et al., 2007) and discarded all fixes with HDOP > 3. Below, we refer to the remaining locations as “valid locations”. For our first objective, we estimated daily (from 00:00 to 24:00) distances traveled by foxes by adding linear distances between successive valid locations, using the adehabitatLT library (Calenge, 2006) in R 3.6.1 (R Core Team, 2019).

For our second objective, we subsampled the dataset with a 30‐s fix interval (30.54 ± 8.09 s) to obtain new datasets with fix intervals > 30 s. For each of the 12 available fox‐days, we simulated six fix intervals (1, 2, 4, 15, 30, and 60 min), corresponding, respectively, to 1,440, 720, 360, 96, 48, and 24 fix attempts/day. The longer fix intervals in this series are commonly used in GPS tracking studies (e.g., Dickie et al., 2016; Evans et al., 2016; Pagano et al., 2018). For each subsampled dataset, the estimated distance traveled was calculated as described above. To test our prediction that daily distance traveled is a logarithmic function of fix interval, we fitted two generalized linear models (GLMs) with daily distance traveled as the response variable, using a gamma distribution (link = “log”) in the R package lme4 (Bates et al., 2015). The explanatory variable was the number of locations in the first model and the logarithm of the number of locations in the second model. We then compared the two models using Akaike information criterion (AIC).

### 2.4 GPS location error

To estimate location error associated to GPS locations, we left from 4 to 9 July 2019 three GPS collars at a fixed location in a representative fox habitat of the study area. Collars 1, 2, and 3 collected 434, 673, and 679 valid GPS locations at a 4‐min fix interval during 31, 46, and 47 consecutive hours, respectively. For each collar, we calculated distances between all GPS locations and their centroid, which we assumed was the true location. The mean ± standard deviation (*SD*) GPS location error was 12.52 ± 67.74 m, 8.02 ± 11.10 m, and 12.98 ± 13.60 m for collars 1, 2, and 3, respectively (giving an overall average of 11.18 ± 30.81 m). The presence of a few outlier GPS locations increased *SD* for collar 1.

To assess the effect of location error on estimated distances traveled by foxes, we introduced simulated errors ranging from 1 to 50 m (1‐m increments) into each of the 96 daily fox tracks obtained with a 4‐min fix interval. We computed 100 iterations for each combination of fox‐day and simulated error. This yielded 5,000 estimated distances traveled for each track, for a total of 480,000 estimated distances (96 daily fox tracks × 50 simulated errors × 100 iterations). Error simulation was done by randomly adding errors in one of the cardinal directions to every valid location. For example, when simulating a 4‐m error to the 20 June track of fox F1, the first location was moved 4 m to the East, the second was moved 4 m to the North, the third was moved 4 m to the East, the fourth was moved 4 m to the South, etc. until all locations had been moved 4 m in a random direction. Distances traveled were estimated by adding linear distances between simulated locations. To evaluate the potential overestimation of traveled distances at the shortest fix interval, we repeated the above simulation for each of the 12 daily fox tracks obtained with a 30‐s fix interval, yielding to a total of 60,000 estimated distances (12 daily fox tracks × 50 simulated errors × 100 iterations). To test our prediction that the relationship between estimated daily distance traveled and simulated location error is exponential, we fitted two generalized linear models (GLMs) with estimated daily distance traveled as the response variable using a gamma distribution (link = “log”). The explanatory variable was the simulated error in the first model and the exponential of the simulated error in the second model. We then compared the two models using AIC. This was done for both the 4‐min and the 30‐s interval datasets.

Results are reported as mean ± *SD*, except if stated otherwise.

## 3 RESULTS

### 3.1 Daily distances traveled

From 5 to 16 July (fix interval = 4 min), we obtained 347 ± 22 successful fixes per fox‐day (fix success rate = 96.4% ± 0.1). HDOP was ≤ 3 in 99.29% of successful fixes (HDOP averaged 1.0 ± 0.3 after removing values > 3); thus, 345 ± 23 valid locations per fox‐day were available for analyses. From 29 July to 2 August (fix interval = 30 s), we obtained 2,858 ± 12 successful fixes per fox‐day (fix success rate = 99.2% ± 0.04). HDOP was ≤ 3 in 98.98% of successful fixes (HDOP averaged 1.1 ± 0.3 after removing values > 3); thus, 2,829 ± 24 valid locations per fox‐day were available for analyses.

**FIGURE 2:**
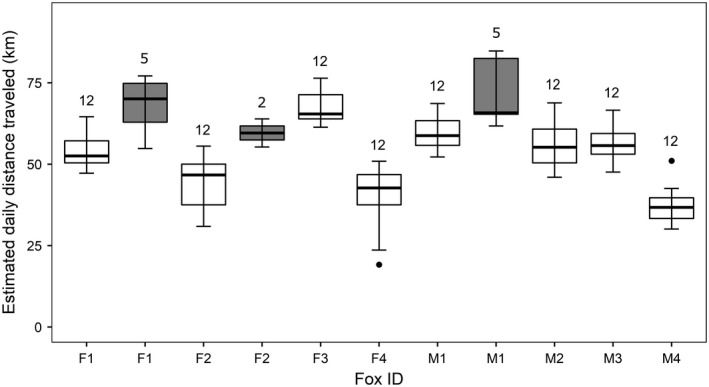
Estimated daily distances traveled by eight arctic foxes GPS‐tracked in 2018 on Bylot Island (Nunavut, Canada). Females F1 to F4 and males M1 to M4 were tracked from 5 to 16 July with a fix interval of 4 min for a total of 96 fox‐days (white boxes). Females F1 and F2 and male M1 were also tracked from 29 July to 2 August at a 30‐s interval, for a total of 12 fox‐days (gray boxes). Boxplots show first quartile, median, and third quartile. Lower and upper whiskers extend, respectively, to the lowest and highest value within the interquartile range multiplied by 1.5. Points represent values outside this range. Numbers on top of boxplots show number of sampling days for each fox

From 5 to 16 July (fix interval = 4 min), the estimated daily distance traveled by foxes was on average 51.88 ± 11.72 km (*n* = 96 fox‐days). We observed individual variation in daily distance traveled, as well as differences explained by fix intervals, as illustrated in Figure 2. The maximum estimate was 76.45 km traveled by F3 on 12 July (354 valid locations obtained out of a potential of 360). The next maximum estimates obtained with a 4‐min fix interval were 75.46 km (F3, 9 July, 340 valid locations) and 75.20 km (F3, 10 July, 357 valid locations). From 29 July to 2 August (fix interval = 30 s), the estimated daily distance traveled by foxes was on average 68.28 ± 9.78 km (*n* = 12 fox‐days), while the maximum estimate was 84.82 km traveled by M1 on 30 July (2,865 valid locations obtained out of a potential of 2,880).

### 3.2 Effect of fix interval on daily distance traveled

**FIGURE 3:**
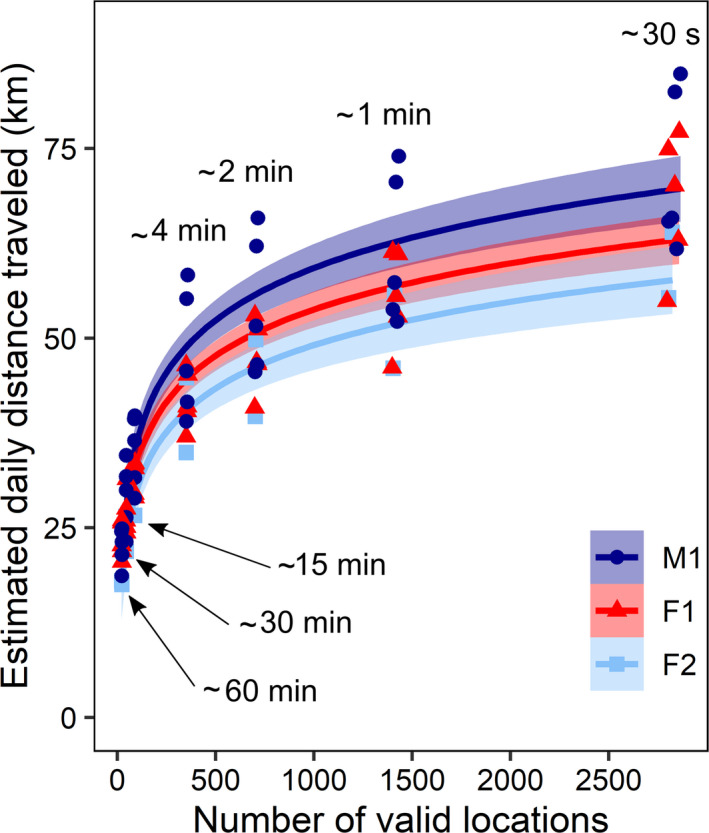
Estimated daily distances traveled by three GPS‐tracked arctic foxes from 29 July to 2 August 2018 on Bylot Island (Nunavut, Canada). Distances are plotted against number of valid locations, ranging from 22 to 2,865 fixes per day (corresponding, respectively, to approximately 60‐min and 30‐s fix intervals). Fix intervals longer than 30 s were subsampled from an original dataset comprising fixes collected at 30‐s intervals. Foxes F1 and M1 were each tracked during 5 days (29 July–2 August), whereas fox F2 was tracked during 2 days (30–31 July). Logarithmic models were fitted for each individual and are represented with their standard errors (colored areas). Results are detailed in Table 2

**FIGURE 4:**
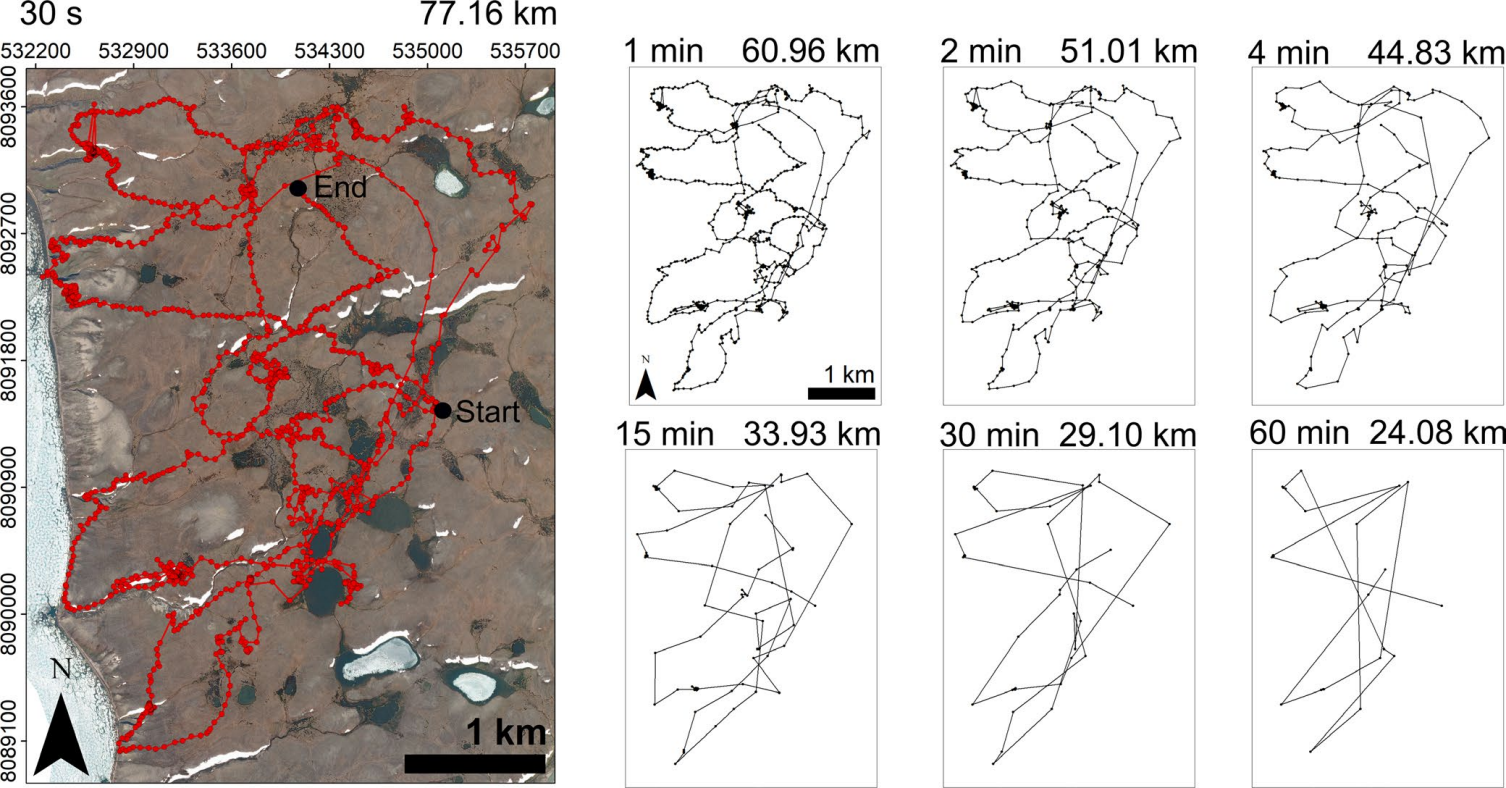
Estimated trajectories and estimated daily distances traveled by arctic fox F1 GPS‐tracked on 31 July 2018 on Bylot Island (Nunavut, Canada). Panels show results obtained with six fix intervals ranging from 60 min to 30 s. Fix intervals longer than 30 s were subsampled from an original dataset comprising fixes collected at 30‐s intervals. Fix interval and estimated distance traveled are shown on the top left and top right of each panel, respectively. The 30‐s panel also gives the UTM coordinates of the study area, as well as the start and end of the track

Estimated daily distance traveled by foxes was a logarithmic function of fix frequency (Figure 3, see Table 2 for detailed results). This logarithmic function outperformed a linear function (∆AIC = 96.19). Daily distances estimated with a 4‐min fix interval were 64.7 ± 4.4% those estimated with a 30‐s fix interval. With a 60‐min fix interval, daily distances were 32.7 ± 5.1% and 50.7 ± 7.7% those estimated with 30‐s and 4‐min fix intervals, respectively. Figure 4 illustrates how estimated trajectories are simplified and estimated daily distances traveled are shortened when fix intervals increase from 30 s to 60 min. A visual analysis of Figure 4 shows a strong decrease of tortuosity when fix interval increases.

**TABLE 2:** Estimated daily distances traveled by three GPS‐tracked arctic foxes from 29 July to 2 August 2018 on Bylot Island (Nunavut, Canada), as a function of the number of valid locations

Number of attempted locations	Number of valid locations (mean ± *SD*)	Corresponding fix interval	Estimated daily distance traveled compared to 30‐s FI (mean ± *SD*) %
2,880	2,829 ± 24	~30 s	100
1,440	1,414 ± 12	~1 min	83.76 ± 2.79
720	707 ± 6	~2 min	73.18 ± 4.13
360	353 ± 3	~4 min	64.67 ± 4.37
96	88 ± 1	~15 min	48.31 ± 3.83
48	45 ± 1	~30 min	40.27 ± 3.50
24	23 ± 1	~60 min	32.68 ± 5.15

Note:

Fix intervals longer than 30 s were subsampled from an original dataset comprising fixes collected at 30‐s intervals. Foxes F1 and M1 were each tracked during 5 days (29 July–2 August), whereas fox F2 was tracked during 2 days (30–31 July). Corresponding number of attempted fixes and fix interval are also given. The relationship between estimated daily distance traveled and number of valid locations is illustrated in Figure 3.

### 3.3 Effect of location error on daily distance traveled

**FIGURE 5:**
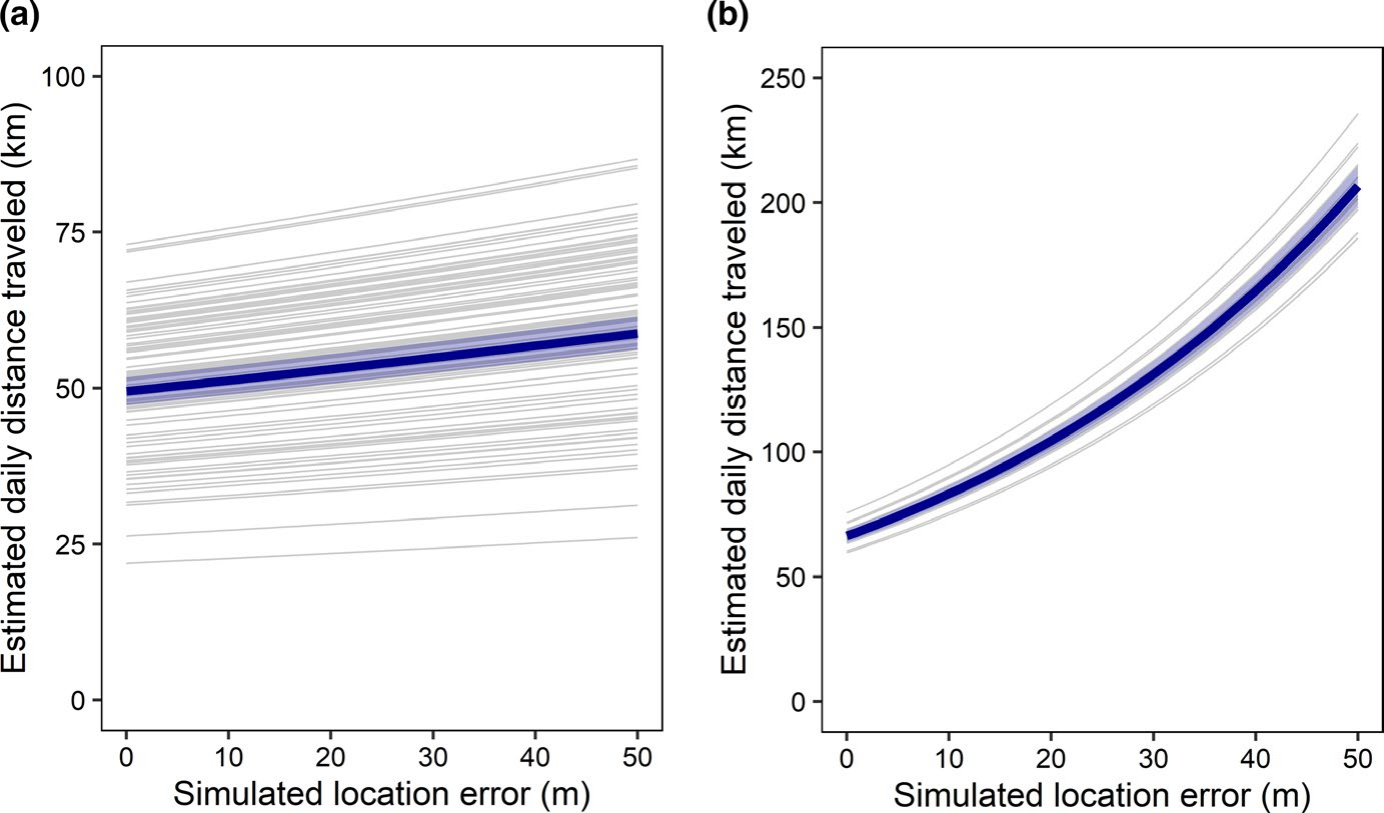
Effect of simulated GPS location error on estimated daily distance traveled by eight arctic foxes tracked with a fix interval of a) 4 min during 96 fox‐days (5–16 July 2018) and b) 30 s during 12 fox‐days (29 July–2 August 2018) on Bylot Island (Nunavut, Canada). Each gray line represents one fox‐day (average of 100 iterations, see Methods), the thick blue line represents the global trend across all fox‐days, and the shaded blue area the corresponding 95% confidence interval

Estimated daily distance traveled by foxes was a linear function of simulated location error (Figure 5). For both fix intervals of 4 min and 30 s, the linear function outperformed the exponential function (∆AIC = 19,324 and ∆AIC = 171,939, respectively). Introducing a 11‐m error into fox tracks at a 4‐min fix interval increased estimated daily distance traveled by only 2.16 ± 1.16% (*n* = 9,600), whereas introducing a 50‐m error led to a 18.07 ± 8.30% increase (Figure 5). However, introducing an 11‐m error into fox tracks at a 30‐s fix interval increased estimated daily distance traveled by 24.20 ± 5.10% (*n* = 1,200).

## 4 DISCUSSION

Our study provides the first detailed report of arctic fox movement rates in their summer territory and, to our knowledge, the first demonstration that a terrestrial mammal can cover daily and routinely such large distances within its territory. Distances traveled daily by arctic foxes averaged 52 km and reached 76 km when estimated through positioning every 4 min. These high movement rates may seem extraordinary for a small (3 kg) terrestrial mammal, yet our results are robust because location error and fix frequency did not significantly bias estimates of traveled distances at 4‐min fix intervals. We first discuss the implications of our results for our knowledge of arctic fox ecology and our understanding of predator–prey relationships. We then evaluate the methodological implications of our research within the field of movement ecology.

### 4.1 Arctic fox ecology and predator–prey relationships

The foraging success of arctic foxes depends on their ability to find vulnerable prey. On Bylot Island during summer, vulnerable prey consist of small mammals found out of their burrow, eggs left unattended or poorly defended, incautious or weakened birds, and animal carcasses. As the availability of such prey is hard to predict in space and time, traveling repeatedly through the territory is critical to maximize foraging success (Eide et al., 2005; Elmhagen et al., 2000). In this context, it is not surprising that arctic foxes travel much higher daily distances than ambush (sit‐and‐wait) predators such as the puma (*Puma concolor*), the Canada lynx (*Lynx canadensis*), or the Eurasian lynx (*Lynx lynx*) (Table 3), which rely on concealment and surprise. How daily movement rates of arctic foxes should compare to those of other mobile predators, particularly canids, is more difficult to envision, but a review of the literature (Table 3) can generate hypotheses despite differences in fix frequency across studies. Gray wolves, coyotes (*Canis latrans*), and kit foxes (*Vulpes macrotis*) may travel daily, on average, 12–22 km when estimated with fix intervals of 15 min to 60 min, with maximum values of 32–70 km obtained with fix intervals of 5–10 min (Table 3). These estimates are lower but not dramatically different from the average movement rates of 15–25 km per day that we obtained with subsampled fix intervals of 15 to 60 min (Figure 3) and from the maximum movement rates of ca. 75 km per day obtained with fix intervals of 4 min. Due to their foraging ecology and habitat characteristics, arctic foxes may thus reside at the greater end of the gradient of movement rates found in mobile predators, or at least in wild canids. It is noteworthy that, as already observed in other canids (e.g., Jedrzejewski et al., 2001; Schlägel et al., 2017), intensive movements within home ranges by arctic foxes may also be related to territory maintenance and patrolling. For example, territorial patrolling and scent‐marking, especially at the borders of the territory, may increase daily ranges in wolves (Jedrzejewski et al., 2001; Zub et al., 2003).

**TABLE 3:** Daily distances traveled by six terrestrial carnivores tracked with GPS or VHF technology

Species	Technique—Location	Daily distance traveled (km)	Fix interval	Reference
Gray wolf (*Canis lupus*)	GPS—Alberta and Saskatchewan (Canada)	70.4 (max) 0.96–70.4 (range)	5 min	Dickie et al. (2016)
Gray wolf (*Canis lupus*)	GPS—British‐Columbia (Canada)	ca. 9 (max monthly average)	20 min or 3 hr	Ehlers et al. (2014)
Gray wolf (*Canis lupus*)	VHF—Białowieża (Poland)	21.6 ± 2.4 (mean ± *SE*)	15 min or 30 min	Theuerkauf et al. (2003)
Gray wolf (*Canis lupus*)	GPS—Alaska (USA)	18.6 ± 0.4 (mean ± *SE*)	60 min	Bryce (2017)
Coyote (*Canis latrans*)	VHF—Durango (Mexico)	16.5 ± 4.9 (mean ± *SD*, males) 12.5 ± 3.5 (mean ± *SD*, females)	60 min	Servín et al. (2003)
Coyote (*Canis latrans*)	VHF—Indiana (USA)	14.2 ± 0.9 (mean ± *SD*)	60 min	Atwood et al. (2004)
Kit fox (*Vulpes macrotis*)	VHF—California (USA)	32 (max)	10 min	Girard (2001)
Puma (*Puma concolor*)	GPS—California (USA)	7.4 ± 2.2 (mean ± *SD*, males) 4.1 ± 0.5 (mean ± *SD*, females)	15 min	Wang et al. (2017)
Puma (*Puma concolor*)	GPS—Patagonia (Chile)	13.4 ± 2.5 (mean ± *SD*) 53 (max)	2 hr	Elbroch and Wittmer (2012)
Canada lynx (*Lynx canadensis*)	GPS—Montana (USA)	7.0 ± 3.2 (mean ± *SD*; females)	30 min	Olson et al. (2011)
Eurasian lynx (*Lynx lynx*)	VHF—Białowieża (Poland)	7.2 ± 5.6 (mean ± *SD*) 0–24.8 (range)	30 min	Jędrzejewski et al. (2002)

Note

Data come from 11 publications containing "daily distance traveled" or "distance traveled" or "daily distance" in their title, abstract, author keywords or Web of Science keywords, and retrieved from Web of Science and Google Scholar in March 2020. Only publications reporting data from individuals having well‐defined home ranges were retained. Reports of daily distances traveled are not standardized and may indicate maximum or average values over varying time intervals, therefore comparisons across species, techniques, or locations must be interpreted with caution. Results of the literature search are representative rather than exhaustive because some relevant publications may not include our searched keywords.

Based on the above, we suggest that future empirical studies should report movement rates obtained at various fix intervals to allow robust comparisons across study systems. Specifically, studies using fix intervals < 15 min should report movement rates obtained through simulation of larger fix intervals (e.g., 60 min, 2, 12 hr) to facilitate comparisons with historic or contemporary data obtained at low fix frequencies (many of such examples are shown in Table 3). Comparing study systems is a necessary step to explain how differences in, for example, habitat, morphology, foraging strategy, season, territory defense needs, and energy requirements affect movement rate across species, populations, and individuals. In this context, dividing daily distance traveled by home range diameter may open interesting avenues for comparing study systems. We estimated (fixed kernel method with a 95% isopleth, analyses not shown) that the size of arctic fox pair summer territories averaged 9.5 ± 1.7 km^2^ (range: 6.8–11.3 km^2^) in our study area during the summer 2018. This corresponds to an average territory diameter of ca. 3.5 km. Our estimated average daily distance of 52 km traveled by arctic foxes thus indicates that a fox could in theory cross its entire territory about 15 times on an average day. This provides a useful indication of movement intensity in this small carnivore and represents a benchmark against which other species can be compared in the future.

The maximum daily distances traveled by territorial arctic foxes in our study area during summer are impressive but not as large as those measured in various seasons for dispersing foxes from the same (>90 km/day; Tarroux et al., 2010) or other populations (112 and 150 km/day; Lehner, 2012 and Fuglei & Tarroux, 2019, respectively). We collected locations at a higher frequency than dispersal studies (which typically collected one or a few fixes per day) so methodological disparities cannot explain the observed difference in daily distance traveled. We rather suggest that the fitness benefits of traveling large daily distances are greater when individuals disperse than when they live in their territory. This could stem from the unique characteristics of arctic fox dispersal, where individuals need to cross vast and inhospitable arctic habitats when searching for a new territory. Interestingly, some predators have been reported to travel greater yearly distances within their territory than during dispersal (Joly et al., 2019). In particular, gray wolves maintaining a territory may travel up to 450 km more than long‐distance dispersing conspecifics over a year (Joly et al., 2019). We do not know, however, how these results translate into maximum distances traveled daily.

Predation impacts prey populations beyond direct consumptive effects (Cresswell, 2008; Teckentrup et al., 2018). Predator movements generate spatial variation in predation risk that can be perceived by prey, which can further respond by modifying their behavior (Gaynor et al., 2019). Prey will for example avoid areas that are highly used by their predators (Simon et al., 2019; Valeix et al., 2009). The distance traveled by predators within their territory could have critical effects on the spatial and temporal distribution of predation risk, since a predator's movement rate strongly determines its encounter rate with prey, which in turn controls its consumption rate (Holling, 1959; Merrill et al., 2010; Pawar et al., 2012). Further research is needed to investigate the importance of distance traveled by predators in predator–prey dynamics (but see Austin et al., 2006; Sperry et al., 2008), as well as its correlation to observed predation rates and the landscape of risk perceived by prey.

Arctic foxes living in a dynamic tundra ecosystem are interesting models to inform subtle, spatially mediated relations linking predators and their prey. Our data were collected in a year of low lemming density in a large goose colony. Fox movement rates could be lower when lemming densities are high, as prey are more abundant and easier to find, but higher outside of the colony, where prey are less abundant. Alternatively, given that high lemming densities induce fox reproduction and thus higher foraging needs, travel rates could increase when lemmings are abundant. Such research context opens many interesting perspectives for exploring the mechanisms explaining apparent competition and apparent mutualism in a system with preferred, alternative and incidental prey sharing a common predator (McKinnon et al., 2013). Furthermore, different lemming or goose densities within each territory could explain interindividual variations in movement rates. Lastly, individual variation in movement rates (Spiegel et al., 2017) could also be explained by consistent among‐individual differences in behavior (i.e., personality, Réale et al., 2007), territory size (Harrison et al., 2015), and foraging strategies (Toscano et al., 2016).

### 4.2 Methodological implications

Whereas the recent affordability of the GPS technology has generalized high location precision in telemetry studies, high location frequency is still rare because tracking devices have a limited battery capacity. We were able to overcome this limitation by using GPS collars equipped with rechargeable batteries and miniature solar panels and by working during summer in the arctic tundra, where 24‐hr daylight and open landscapes favor battery charging. Open landscapes also eased transmission of data between GPS satellites and collars, and between collars and base stations, which avoided waste of energy due to failed attempts when data transit in and out of collars. Our study thus provides another example of the technological revolution benefiting movement ecology (Hofman et al., 2019; Nathan, 2008; Wilmers et al., 2015).

As predicted, estimated daily distance traveled was a logarithmic function of sampling frequency and was thus largely underestimated at low fix frequencies. A major contribution of our work is to provide empirical data that reflected very well true animal trajectories. Based on this, we stress again the need for field studies to give proper attention to the effect of fix interval on estimated movement rates. We, however, acknowledge that study designs can also reflect objectives and constraints other than obtaining unbiased estimates of movement rates (Williams et al., 2020). Fix interval should match the spatiotemporal scales of both individual movements (Jerde & Visscher, 2005) and tested hypotheses, while optimizing battery life (Hofman et al., 2019) as this determines the sampling period.

According to the literature, high‐resolution spatial analyses permitted by high fix frequency allow detailed testing of hypotheses regarding space use, foraging ecology, and foraging behavior of animals. For example, fix interval affects estimates of home range size (Börger et al., 2006; Mills et al., 2006; Mitchell et al., 2019) and selection coefficients (Frair et al., 2004; Johnson & Gillingham, 2008) when investigating animal space use and habitat selection. Since habitat selection is a hierarchical process that is scale‐dependent (Johnson, 1980), measuring habitat selection at a fine scale can reveal selected landscape features that would not be identified at a coarser spatiotemporal resolution (e.g., Bischof et al., 2019; Zeller et al., 2016). For instance, intensive GPS bursts revealed linear feature tracking by red foxes (*Vulpes vulpes*) in a mosaic landscape, a behavior restricted in time and space that would have remained hidden if less frequent fixes had been used (Bischof et al., 2019). Lastly, fine‐scale movement data, from which one can accurately derive a consumer's relative velocity, can also be used to incorporate foraging strategies into mechanistic models studying trophic interactions (Pawar et al., 2012).

Our 11‐m estimate of average GPS precision matches or surpasses estimates commonly found in the literature (Frair et al., 2010). The open tundra landscapes likely explain this good performance. However, as in most other studies (but see Christin et al., 2015), we estimated the precision of static collars even though moving animals were studied, so it is possible that we underestimated true errors. Future research should strive to estimate precision of moving GPS devices, especially when studying fast moving animals.

Contrary to our prediction, estimated daily distance traveled by foxes was a near‐linear, rather than an exponential function of simulated location error. At a fix interval of 4 min, a location error of 11 m increased our estimation of daily distance traveled by only 2% on average. This result is reassuring because it indicates a low risk that estimated daily distances traveled by animals are severely inflated by GPS location error at this fix interval. However, although our GPS devices were precise enough to prevent a biologically meaningful inflation of movement rates at a 4‐min fix interval, precision was too low to prevent a substantial inflation at 30‐s intervals. Daily distances traveled estimated at 30‐s fix intervals were clearly overestimated. Given that GPS units keep track of visible satellite signals and thus become more precise when fix interval is < 7 s, it would be interesting to test the effect of fix intervals < 7 s on estimated daily distance traveled. We also note that one should be careful not to extrapolate our results to times of the year when foxes have lower movement rates, to species with much lower velocities than arctic foxes, or to studies involving less precise telemetry techniques, such as the Argos system (Jerde & Visscher, 2005).

## 5 CONCLUSIONS

Using GPS collars equipped with solar panels, we demonstrated that arctic foxes are highly mobile predators that travel extensive distances within their summer territories. Our results have important implications for our understanding of the foraging ecology of small mobile predators and the risk incurred by their prey. Our study also stresses the need to use an optimal sampling frequency when measuring animal movement, since fixes obtained at long intervals greatly underestimate movement rate of highly mobile animals due to the linear estimation of tortuous movements, whereas fixes obtained at very short intervals can lead to overestimation due to location error. Our empirical assessment of the effects of fix interval and location error on estimated movement rates can guide the design and interpretation of studies on the movement ecology of small opportunistic foragers. Improving tracking technology with smaller devices, increased battery life, and increased location precision can greatly improve our ability to accurately measure animal movement in a diversity of species, hence improving our understanding of the ecology and evolution of organisms.

## CONFLICT OF INTEREST

The authors declare that they have no conflict of interests.

## AUTHOR CONTRIBUTIONS

**Marie‐Pier Poulin:** Conceptualization (equal); formal analysis (lead); visualization (lead); writing – original draft (equal); writing – review and editing (lead). **Jeanne Clermont:** Conceptualization (equal); formal analysis (supporting); writing‐original draft (equal); writing – review and editing (supporting). **Dominique Berteaux:** Conceptualization (equal); funding acquisition (lead); supervision (lead); writing – original draft (equal); writing – review and editing (supporting).

## Data Availability

All arctic fox GPS data are available through the Movebank Data Repository at Berteaux, D. 2020, Arctic fox Bylot—GPS tracking, Movebank Study ID 1241071371.
